# Supplementary material for: Sepsis endotypes identified by host gene expression across global cohorts
Source: Commun Med (Lond). 2024 Jun 18;4:120. doi: 10.1038/s43856-024-00542-7 (PMC11189468; doi:10.1038/s43856-024-00542-7)
Supplement: Supplementary file 2 — Supplementary Information [file 43856_2024_542_MOESM2_ESM.pdf]

Sepsis Endotypes Identified by Host Gene Expression Across Global Cohorts.

Supplementary Information

AUTHORS

Josh G. Chenoweth[1], Joost Brandsma[1], Deborah A. Striegel[1], Pavol Genzor[1], Elizabeth Chiyka[1], Paul W. Blair[1], Subramaniam Krishnan[1], Elliot Dogbe[2], Isaac Boakye[3], Gary B. Fogel[4], Ephraim L. Tsalik[5, 12], Christopher W. Woods[5], Alex Owusu-Ofori[2,6], Chris Oppong[7], George Oduro[7], Te Vantha[8], Andrew G. Letizia[9], Charmagne G. Beckett[10], Kevin L. Schully[11], Danielle V. Clark[1].

AFFILIATIONS

[1]Austere environments Consortium for Enhanced Sepsis Outcomes (ACESO), The Henry M. Jackson Foundation for the Advancement of Military Medicine, Inc., Bethesda, Maryland, USA. [2]Laboratory Services Directorate, KATH, Kumasi, Ghana. [3]Research and Development Unit, KATH, Kumasi, Ghana. [4]Natural Selection, Inc. San Diego, CA, USA. [5]Center for Infectious Disease Diagnostics and Innovation, Department of Medicine, Duke University School of Medicine, Durham, NC, USA. [6]Department of Clinical Microbiology, Kwame Nkrumah University of Science and Technology (KNUST), Ghana. [7]Accident and Emergency Department, KATH, Kumasi, Ghana. [8]Takeo Provincial Referral Hospital, Takeo, Cambodia. [9]Naval Medical Research Unit EURAFCENT Ghana detachment, Accra, Ghana. [10]Naval Medical Research Command Infectious Diseases Directorate, Silver Spring, MD, USA. [11]Austere environments Consortium for Enhanced Sepsis Outcomes (ACESO), Biological Defense Research Directorate, Naval Medical Research Command-Frederick, Ft. Detrick, MD, USA. [12]Present address: Danaher Diagnostics, Washington, DC., USA.

Corresponding Author: Josh G. Chenoweth; jchenoweth@aceso-sepsis.org

Supplementary Methods

Retrieval and analysis of public sepsis gene sets.

To compare our observed gene expression to published studies, we reviewed and selected sepsis-related literature that identifies different *endotype* patterns. We then retrieved publicly available expression data along with relevant sample metadata using the information provided in publications. We then reproduced reported differential analyses and selected only the genes in 8- and 13-gene sets reported in our study. The expression trend of our set of genes and calculated $\log_2$ fold change values from public data were plotted in a heatmap and compared (Supplementary Fig. 6). The public data sets used for this analysis included: Reyes *et.al.*, 2020 (PMID: 32066974; data sets: Broad Single Cell Portal SCP548), Davenport *et.al.*, 2016 (PMID: 26917434; data sets: E-MTAB-4421, E-MTAB-4451), Cazalis *et.al.*, 2014 (PMID: 26215705; data set: GSE57065), Tsalik *et.al.*, 2014 (PMID: 25538794; data set: GSE63042).

a

Table1: Demographics

		Female	Male	Total N (%)	p-value
**Entire Cohort**	N (%)	211 (43%)	283 (57%)	494	
	Mean Age (SD)	50.35 (17.10)	50.58 (17.40)		0.8816
	Median Age (IQR)	48 (37-63.50	50 (36-63)		
	*d28 mortality (%)	40 (19%)	45 (16%)	85 (17%)	0.3741
**Cambodia**	N (%)	52 (31%)	115 (69%)	167	
	Mean Age (SD)	54.58 (16.62)	47.69 (16.05)		0.0139
	Median Age (IQR)	54 (45-70)	48 (35-60.50		
	*d28 mortality (%)	8 (15%)	13 (11%)	21 (13%)	0.0126
**Duke**	N (%)	72 (44%)	92 (56%)	164	
	Mean Age (SD)	49.13(17.34)	54.54 (17.46)		0.0456
	Median Age (IQR)	48 (35-63)	54.5 (46-65.5)		
	*d28 mortality (%)	3 (4%)	10 (11%)	13 (8%)	0.0351
**Ghana**	N (%)	82 (52%)	76 (48%)	158	
	Mean Age (SD)	48.81(16.93)	50.16 (18.54)		0.6335
	Median Age (IQR)	45 (35.3-61)	50 (35-65)		
	*d28 mortality (%)	29 (35%)	22 (29%)	51 (32%)	0.6838

N = Count or number of patients

SD = Standard Deviation

IQR = Inner Quartile Range (25% - 75% of data)

d28 mortality = Number of patients that died at day 28

* Wilcoxon rank sum test results. All other results are from Welch Two Sample t-test

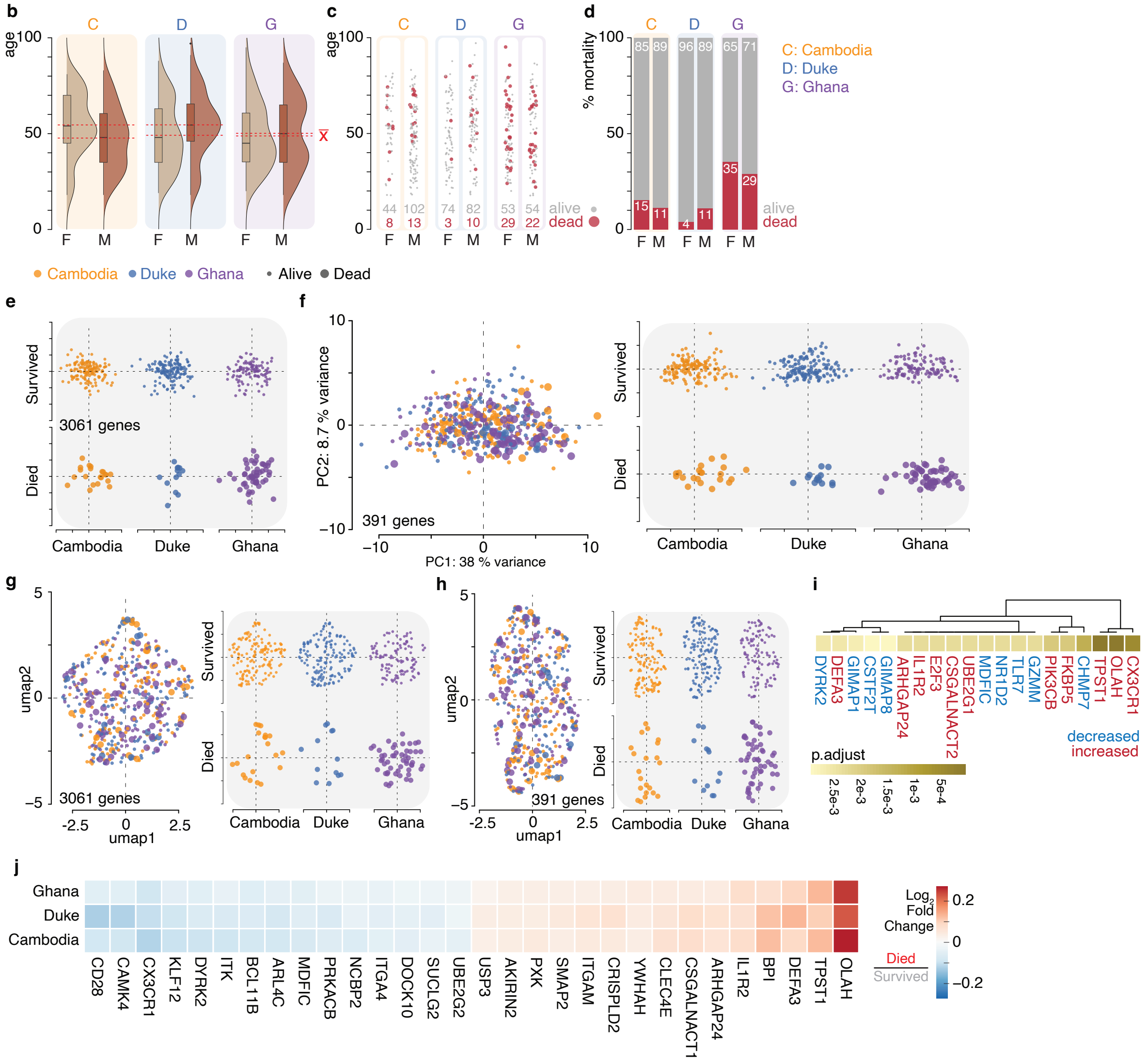

Supplementary Fig. 1: Experimental design, demographics, and initial modeling

a A table of age and mortality comparisons of gender for the entire study cohort and each study site (Cambodia - light orange, Duke - light blue, Ghana - light purple). Red values indicate significant differences (p-value =< 0.05). **b, c** Distribution of participant age across by gender for each study site and (**d**) summary of 28-day mortality (F: female - light tan; M: male - light brown). Boxplots show medians and quartiles, and red dotted lines indicate means (x). Red spheres and bar fill indicate patients that died. **e** Principal component analysis (PCA) of 3061 genes for 28-day mortality across three different sites. **f** PCA of 391 selected genes with relevance to mortality for the entire cohort and by site and 28-day mortality. Uniform Manifold Approximation and Projection for Dimension Reduction (UMAP) analysis of the (**g**) 3061 genes and (**h**) 391 genes are shown for the entire study cohort, by site and 28-day mortality (Point color indicates site, and point size indicates mortality; Cambodia - orange, Duke - blue, Ghana - purple). **i** Significant p-value for the top 10 increased (red labels) and top 10 decreased (blue labels) genes in differential gene expression comparing 28-day mortality for the entire cohort (same as Figure 1F). **j** Heatmap depicting a subset of 28-day mortality significant genes (Figure 1f) that are also significantly different across each of the study sites.

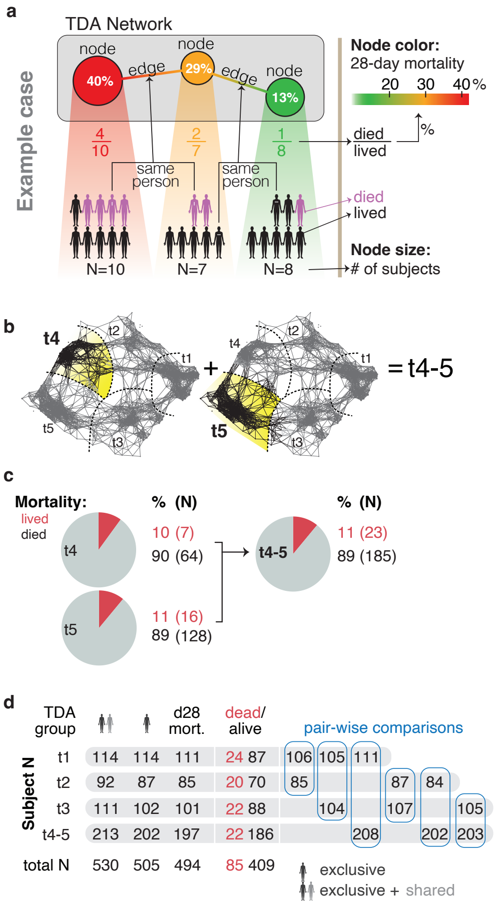

Supplementary Fig. 2: Topological data analysis (TDA) of the sepsis patient cohort

a Hypothetical example of the Topological Data Analysis (TDA) network. This method groups similar patients into *nodes* (groups of individuals with similar patterns of gene expression) that are connected by *edges* (based on individuals shared between nodes), that together form a network. Node size is determined by the total number of contained individuals. Each node is colored by the percent (%) 28-day mortality of individuals contained in the node. **b** The TDA group t4-5 was generated by combining two smaller groups, t4 and t5, with similar mortality rates and a smaller number of subjects. **c** Mortality rate (%) and patient counts (N) in TDA groups t4 and t5 and new group t4-5 (yellow - highlights TDA subgroup; red - indicates mortality). **d** Table summarizing patient count in each TDA group used for multiple subsequent analyses throughout the study.

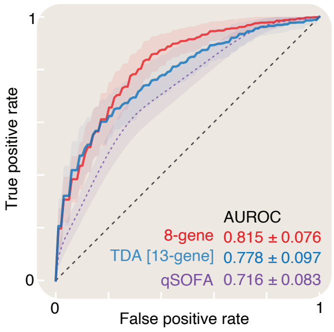

Supplementary Fig. 3: TDA group-specific genes have predictive power

Comparison of the predictive power of different models throughout the study. Receiver operating characteristic (ROC) curves showing the performance of prognostic classifiers including the 8-gene model (red) based on the entire cohort versus quick sepsis-related organ failure assessment (qSOFA) score (purple), and TDA-stratified model (blue). Lighly shaded area surrounding the curves corresponds to 0.5 standard deviations. All modeled curves were generated with repeated stratified k-fold cross-validation described in the methods.

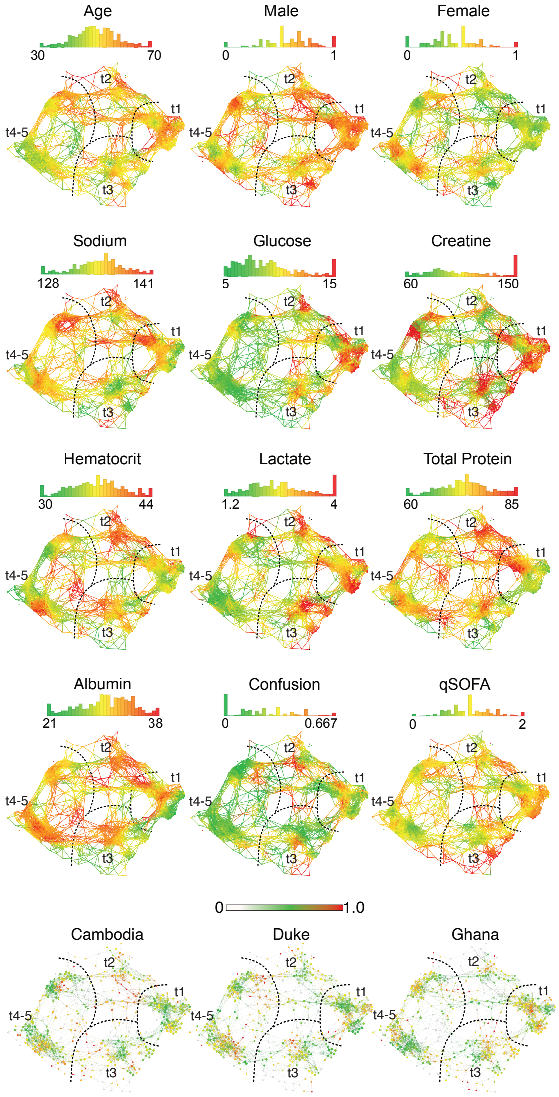

Supplementary Fig. 4: Clinical and laboratory inspection of the global sepsis cohort

TDA overlays of significant demographics, clinical measurements, and symptoms for the entire study cohort. Approximate TDA group assignments are indicated by dotted lines. Colors depict the range of represented data or a fraction of the total. Red and green indicate the maximum and minimum values respectively, while yellow represents the middle of the gradient.

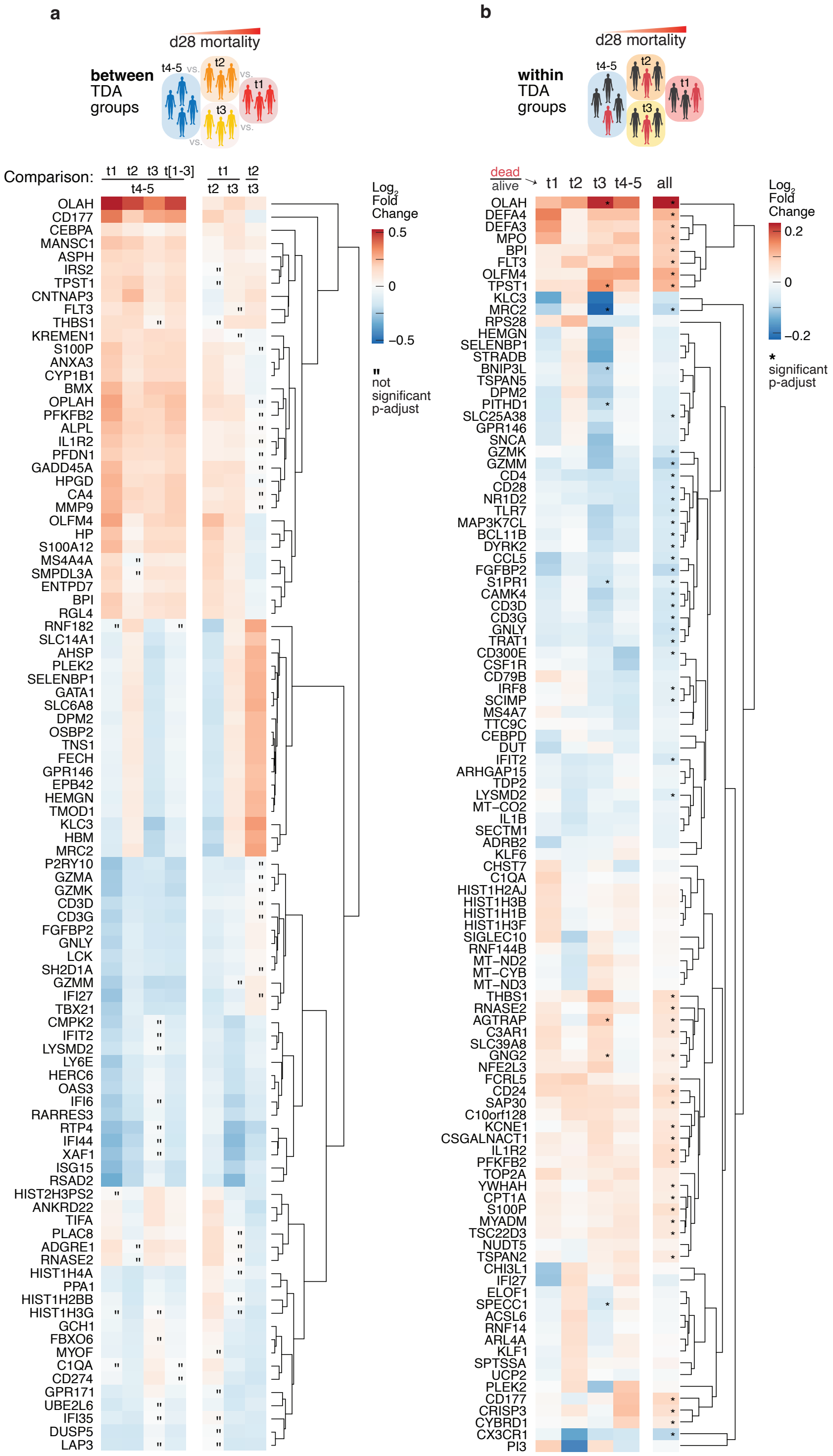

a
d28 mortality
between
TDA
groups
t4-5
t2
t3
t1
vs.
Comparison:
t1
t2
t3
t[1-3]
t4-5
t1
t2
t2
t3
t3
Log2 Fold Change
0.5
0
−0.5
" not significant p-adjust
OLAH
CD177
CEBPA
MANSC1
ASPH
IRS2
TPST1
CNTNAP3
FLT3
THBS1
KREMEN1
S100P
ANXA3
CYP1B1
BMX
OPLAH
PFKFB2
ALPL
IL1R2
PFDN1
GADD45A
HPGD
CA4
MMP9
OLFM4
HP
S100A12
MS4A4A
SMPDL3A
ENTPD7
BPI
RGL4
RNF182
SLC14A1
AHSP
PLEK2
SELENBP1
GATA1
SLC6A8
DPM2
OSBP2
TNS1
FECH
GPR146
EPB42
HEMGN
TMOD1
KLC3
HBM
MRC2
P2RY10
GZMA
GZMK
CD3D
CD3G
FGFBP2
GNLY
LCK
SH2D1A
GZMM
IFI27
TBX21
CMPK2
IFIT2
LYSMD2
LY6E
HERC6
OAS3
IFI6
RARRES3
RTP4
IFI44
XAF1
ISG15
RSAD2
HIST2H3PS2
ANKRD22
TIFA
PLAC8
ADGRE1
RNASE2
HIST1H4A
PPA1
HIST1H2BB
HIST1H3G
GCH1
FBXO6
MYOF
C1QA
CD274
GPR171
UBE2L6
IFI35
DUSP5
LAP3
b
d28 mortality
within
TDA
groups
t4-5
t2
t3
t1
dead
alive
t1
t2
t3
t4-5
all
Log2 Fold Change
0.2
0
−0.2
* significant p-adjust
OLAH
DEFA4
DEFA3
MPO
BPI
FLT3
OLFM4
TPST1
KLC3
MRC2
RPS28
HEMGN
SELENBP1
STRADB
BNIP3L
TSPAN5
DPM2
PITHD1
SLC25A38
GPR146
SNCA
GZMK
GZMM
CD4
CD28
NR1D2
TLR7
MAP3K7CL
BCL11B
DYRK2
CCL5
FGFBP2
S1PR1
CAMK4
CD3D
CD3G
GNLY
TRAT1
CD300E
CSF1R
CD79B
IRF8
SCIMP
MS4A7
TTC9C
CEBPD
DUT
IFIT2
ARHGAP15
TDP2
LYSMD2
MT−CO2
IL1B
SECTM1
ADRB2
KLF6
CHST7
C1QA
HIST1H2AJ
HIST1H3B
HIST1H1B
HIST1H3F
SIGLEC10
RNF144B
MT−ND2
MT−CYB
MT−ND3
THBS1
RNASE2
AGTRAP
C3AR1
SLC39A8
GNG2
NFE2L3
FCRL5
CD24
SAP30
C10orf128
KCNE1
CSGALNACT1
IL1R2
PFKFB2
TOP2A
YWHAH
CPT1A
S100P
MYADM
TSC22D3
NUDT5
TSPAN2
CHI3L1
IFI27
ELOF1
SPECC1
ACSL6
RNF14
ARL4A
KLF1
SPTSSA
UCP2
PLEK2
CD177
CRISP3
CYBRD1
CX3CR1
PI3

Supplementary Fig. 5: Differential Gene Expression within and between TDA groups

a The top fifteen most significantly changed genes (combined from each comparison indicated) between different TDA groups. All changes are statistically significant (p.adjust ≤ 0.05) unless annotated by a quote symbol. **b** Top fifteen most significantly changed genes within TDA groups comparing patients based on 28-day mortality. Only changes annotated by a star * are statistically significant (p.adjust ≤ 0.05). Heatmaps show $\log_2$ fold change values and the genes were grouped using unsupervised hierarchical clustering. The TDA groups were colored and shaded as follows: t4-5 – blue; t3 – yellow; t2 - orange; t1 – red.

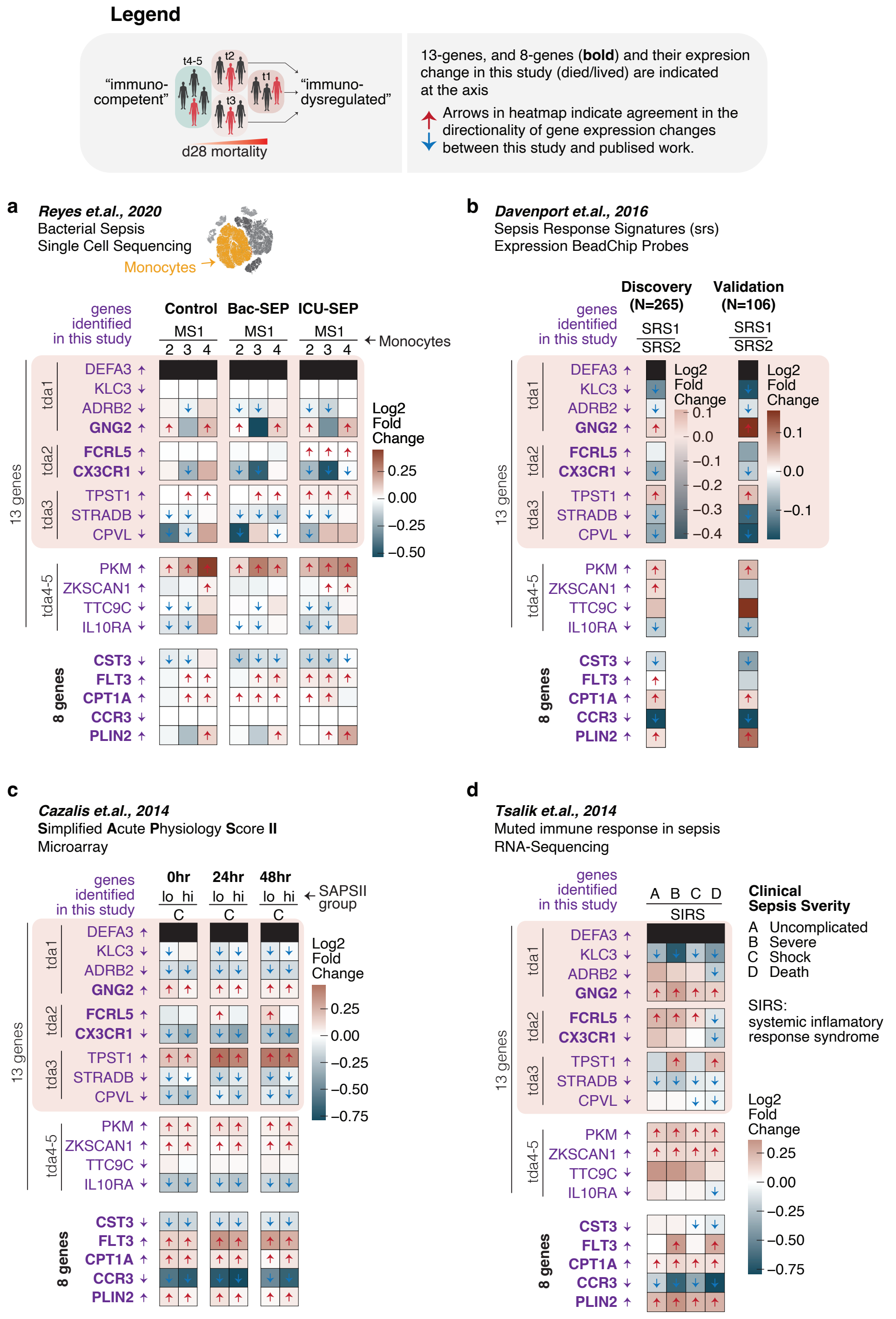

Legend
t4-5
t2
t1
t3
"immuno-competent"
"immuno-dysregulated"
d28 mortality
13-genes, and 8-genes (bold) and their expresion change in this study (died/lived) are indicated at the axis
Arrows in heatmap indicate agreement in the directionality of gene expression changes between this study and publised work.
a
Reyes et.al., 2020
Bacterial Sepsis
Single Cell Sequencing
Monocytes
genes identified in this study
Control
Bac-SEP
ICU-SEP
MS1
2 3 4
Monocytes
13 genes
8 genes
tda1
tda2
tda3
tda4-5
DEFA3
KLC3
ADRB2
GNG2
FCRL5
CX3CR1
TPST1
STRADB
CPVL
PKM
ZKSCAN1
TTC9C
IL10RA
CST3
FLT3
CPT1A
CCR3
PLIN2
Log2 Fold Change
0.25
0.00
−0.25
−0.50
b
Davenport et.al., 2016
Sepsis Response Signatures (srs)
Expression BeadChip Probes
Discovery (N=265)
Validation (N=106)
SRS1
SRS2
Log2 Fold Change
0.1
0.0
−0.1
−0.2
−0.3
−0.4
c
Cazalis et.al., 2014
Simplified Acute Physiology Score II
Microarray
0hr
24hr
48hr
lo hi
C
SAPSII group
Log2 Fold Change
0.25
0.00
−0.25
−0.50
−0.75
d
Tsalik et.al., 2014
Muted immune response in sepsis
RNA-Sequencing
A B C D
SIRS
Clinical Sepsis Sverity
A Uncomplicated
B Severe
C Shock
D Death
SIRS: systemic inflamatory response syndrome
Log2 Fold Change
0.25
0.00
−0.25
−0.50
−0.75

Supplementary Fig. 6: Activity of 8 and 13 genes is recapitulated in published sepsis cohorts

To evaluate the activity of the 8- and 13-gene sets and further validate their use, we analyzed their expression in diverse sepsis study cohorts previously described in the literature. Pressence of an arrow inside colored tiles indicates agreement of directionality of expression observed by us and by previously published work. Red color indicate increase while blue corresponds to decrease. The majority of our genes followed the same directionality of expression change in other sepsis study cohorts. **a** Reyes et.al., 2020 study is a single-cell sequencing study of immune signatures that identifies MS1 class of monocytes as markers of severe sepsis. **b** Davenport et.al., 2016 uses expression bead chips array to identify and validate two sepsis response signature groups (SRS1 & SRS2) related to dysregulation of the immune system and increased sepsis mortality. **c** Cazalis et.al., 2014 study uses microarray measurements to describe gene expression differences between high and low simplified acute physiology score II (SAPSII) scores in sepsis. **d** Tsalik et.al., 2014 study uses whole blood RNA sequencing to show that immune competence is associated with better survival and a dysregulated immune response is associated with higher mortality.
