# Supplementary material for: Sepsis endotypes identified by host gene expression across global cohorts
Source: Commun Med (Lond). 2024 Jun 18;4:120. doi: 10.1038/s43856-024-00542-7 (PMC11189468; doi:10.1038/s43856-024-00542-7)
Supplement: Supplementary file 3 — Description of Additional Supplementary Files [file 43856_2024_542_MOESM3_ESM.pdf]

File Name: **Supplementary Data 1**

Description: The spreadsheet file with seven sheets (tables) containing: a legend describing independent sheets; a summary breakdown of the study cohort demographics; a list of 391 significantly changed genes related to 28-day mortality; a list of 50 genes identified by MRMR; a list of 8 genes used for modeling; a list of 13 TDA-related genes used for modeling; table of genes that are significantly changes across three testing sites.

File Name: **Supplementary Data 2**

Description: The spreadsheet file with twelve sheets (tables) containing: a legend describing independent sheets; results of ANOVA analysis of clinical variables for each TDA group; results of a t-test comparing clinical features between TDA groups; results of differential gene expression (DGE) analysis withing TDA groups considering 28-day mortality; results of DGE analysis between different TDA groups; results of gene set enrichment analysis (GSEA) using Hallmark pathways for entire cohort considering 28-day mortality; results of GSEA using Hallmark pathways between TDA groups; results of GSEA using Hallmark pathways within TDA groups considering 28-day mortality; results of comparative analysis with Reyes et.al. 2020; results of comparative analysis with Cazalis et.al., 2014; results of comparative analysis with Davenport et.al., 2016; results of comparative analysis with Tsalik et.al., 2014
