# Supplementary material for: Sepsis endotypes identified by host gene expression across global cohorts
Source: Commun Med (Lond). 2024 Jun 18;4:120. doi: 10.1038/s43856-024-00542-7 (PMC11189468; doi:10.1038/s43856-024-00542-7)
Supplement: Supplementary file 6 — Reporting Summary [file 43856_2024_542_MOESM6_ESM.pdf]

nature portfolio

Corresponding author(s): Josh Chenoweth

Last updated by author(s): Oct 10, 2023

Reporting Summary

Nature Portfolio wishes to improve the reproducibility of the work that we publish. This form provides structure for consistency and transparency in reporting. For further information on Nature Portfolio policies, see our Editorial Policies and the Editorial Policy Checklist.

Statistics

For all statistical analyses, confirm that the following items are present in the figure legend, table legend, main text, or Methods section.

n/a	Confirmed	
☐	☒	The exact sample size (n) for each experimental group/condition, given as a discrete number and unit of measurement
☒	☐	A statement on whether measurements were taken from distinct samples or whether the same sample was measured repeatedly
☒	☐	The statistical test(s) used AND whether they are one- or two-sided<br>*Only common tests should be described solely by name; describe more complex techniques in the Methods section.*
☐	☒	A description of all covariates tested
☐	☒	A description of any assumptions or corrections, such as tests of normality and adjustment for multiple comparisons
☐	☒	A full description of the statistical parameters including central tendency (e.g. means) or other basic estimates (e.g. regression coefficient) AND variation (e.g. standard deviation) or associated estimates of uncertainty (e.g. confidence intervals)
☐	☒	For null hypothesis testing, the test statistic (e.g. F, t, r) with confidence intervals, effect sizes, degrees of freedom and P value noted<br>*Give P values as exact values whenever suitable.*
☒	☐	For Bayesian analysis, information on the choice of priors and Markov chain Monte Carlo settings
☒	☐	For hierarchical and complex designs, identification of the appropriate level for tests and full reporting of outcomes
☒	☐	Estimates of effect sizes (e.g. Cohen's d, Pearson's r), indicating how they were calculated

Our web collection on statistics for biologists contains articles on many of the points above.

Software and code

Policy information about availability of computer code

Data collection	No software was used for data collection.
Data analysis	All the code used for analysis is deposited at github (https://github.com/HJF-ACESO/Sepsis_One) and will be deposited in Zedono upon publication.

For manuscripts utilizing custom algorithms or software that are central to the research but not yet described in published literature, software must be made available to editors and reviewers. We strongly encourage code deposition in a community repository (e.g. GitHub). See the Nature Portfolio guidelines for submitting code & software for further information.

Data

Policy information about availability of data

All manuscripts must include a data availability statement. This statement should provide the following information, where applicable:

- Accession codes, unique identifiers, or web links for publicly available datasets
- A description of any restrictions on data availability
- For clinical datasets or third party data, please ensure that the statement adheres to our policy

To protect patient confidentiality, the subject level RNA sequencing and clinical metadata are only available via restricted access as patients did not consent for the public release of their data. Investigators interested in accessing subject level data should contact the corresponding authors at the addresses accompanying this manuscript. Supplementary Data 1 and 2 include results of the data analysis used to generate the figures included in the manuscript.

Human research participants

Policy information about studies involving human research participants and Sex and Gender in Research.

Reporting on sex and gender	Sex information was collected as a part of the study protocol described previously in Blair PW, Mehta R, Oppong CK, et alScreening tools for predicting mortality of adults with suspected sepsis: an international sepsis cohort validation study BMJ Open 2023;13:e067840. doi: 10.1136/bmjopen-2022-067840<br><br>Sex information was not considered on individual basis or to draw major conclusions. Any sex-based analyses were performed solely to describe the demographic makeup of each site and an entire study cohort. Any significant sex-differences in this study were noted in the main and supplementary information.
Population characteristics	Various study population characteristics were collected as a part of the study protocol described previously in Blair PW, Mehta R, Oppong CK, et al Screening tools for predicting mortality of adults with suspected sepsis: an international sepsis cohort validation studyBMJ Open 2023;13:e067840. doi: 10.1136/bmjopen-2022-067840<br><br>The primary focus of this study was overall mortality and day 28 mortality metrics.
Recruitment	Entire study cohort in this study has been previously described in Blair PW, Mehta R, Oppong CK, et al Screening tools for predicting mortality of adults with suspected sepsis: an international sepsis cohort validation study BMJ Open 2023;13:e067840. doi: 10.1136/bmjopen-2022-067840
Ethics oversight	Study protocols were approved by the Naval Medical Research Center (NMRC) Institutional Review Board (IRB) (Cambodia sepsis study # NMRC.2013.0019; Ghana sepsis study # NMRC.2016.0004-GHA; Duke sepsis study (Duke # PRO00054849) in compliance with all applicable Federal regulations governing the protection of human subjects as well as host country IRBs.<br><br>The study protocol in Cambodia was approved by the Cambodian National Ethics Committee for Health Research (NECHR). The protocol in Ghana was approved by the Committee on Human Research, Publication and Ethics (CHRPE) at Kwame Nkrumah University of Science & Technology. All procedures were in accordance with the ethical standards of the Helsinki Declaration of the World Medical Association. All patients, or their legally authorized representatives, provided written informed consent.

Note that full information on the approval of the study protocol must also be provided in the manuscript.

Field-specific reporting

Please select the one below that is the best fit for your research. If you are not sure, read the appropriate sections before making your selection.

☒ Life sciences ☐ Behavioural & social sciences ☐ Ecological, evolutionary & environmental sciences

For a reference copy of the document with all sections, see nature.com/documents/nr-reporting-summary-flat.pdf

Life sciences study design

All studies must disclose on these points even when the disclosure is negative.

Sample size	Sample size was determined by considering all consented subject described in Blair PW, Mehta R, Oppong CK, et al Screening tools for predicting mortality of adults with suspected sepsis: an international sepsis cohort validation study BMJ Open 2023;13:e067840. doi: 10.1136/bmjopen-2022-067840
Data exclusions	Of the total 505 original study participants, for the bulk of the analyses we excluded 11 subjects that were lacking information on the mortality outcome.
Replication	No replicate RNA-Sequencing experiments were carried out due to limited amounts of the collected blood and to not cause undue burden to study participants.
Randomization	No randomization strategy of the subjects was employed for this study. If subjects were grouped for analyses it was according to their site of origin or according to clinical outcomes related to mortality wherever applicable.
Blinding	Blinding strategy was not applicable to this study because of its observational and descriptive design.

Reporting for specific materials, systems and methods

We require information from authors about some types of materials, experimental systems and methods used in many studies. Here, indicate whether each material, system or method listed is relevant to your study. If you are not sure if a list item applies to your research, read the appropriate section before selecting a response.

Materials & experimental systems

n/a	Involved in the study
☒	☐ Antibodies
☒	☐ Eukaryotic cell lines
☒	☐ Palaeontology and archaeology
☒	☐ Animals and other organisms
☒	☐ Clinical data
☒	☐ Dual use research of concern

Methods

n/a	Involved in the study
☒	☐ ChIP-seq
☒	☐ Flow cytometry
☒	☐ MRI-based neuroimaging
